# Redox Balance Correlates with Nutritional Status among Patients with End-Stage Renal Disease Treated with Maintenance Hemodialysis

**DOI:** 10.1155/2019/6309465

**Published:** 2019-09-05

**Authors:** Małgorzata Maraj, Beata Kuśnierz-Cabala, Paulina Dumnicka, Katarzyna Gawlik, Dorota Pawlica-Gosiewska, Agnieszka Gala-Błądzińska, Anna Ząbek-Adamska, Piotr Ceranowicz, Marek Kuźniewski

**Affiliations:** ^1^Department of Physiology, Jagiellonian University Medical College, Grzegórzecka 16 St., Krakow 31-531, Poland; ^2^Department of Diagnostics, Chair of Clinical Biochemistry, Jagiellonian University Medical College, Kopernika 15A St., Krakow 31-501, Poland; ^3^Department of Medical Diagnostics, Faculty of Pharmacy, Jagiellonian University Medical College, Medyczna 9 St., Krakow 30-688, Poland; ^4^Dialysis Therapy Centre, St. Queen Jadwiga Clinical District Hospital No. 2, Lwowska 60 St., Rzeszów 35-301, Poland; ^5^Faculty of Medicine, Rzeszow University, Kopisto Ave. 2a, Rzeszów 35-310, Poland; ^6^Diagnostic Department, University Hospital, Kopernika 15B St., Krakow 31-501, Poland; ^7^Chair and Department of Nephrology, Jagiellonian University Medical College, Kopernika 15C St., Krakow 31-501, Poland

## Abstract

Over 50% of end-stage renal disease (ESRD) patients die of cardiovascular disease. ESRD patients treated with maintenance hemodialysis are repeatedly exposed to oxidative stress. The aim of the study was to find the relationship between lifestyle factors, nutritional status, calcium-phosphate metabolism, and selected redox parameters such as glutathione peroxidase (GPx), glutathione reductase (GR), superoxide dismutase (SOD), uric acid (UA), and total antioxidant capacity expressed as ferric reducing antioxidant power (FRAP). The study included 97 ESRD hemodialysis patients and 42 controls with no renal disease. Patients were asked to complete a questionnaire which gathered information on their physical activity, hours of sleep, smoking, and frequency of fruit and vegetable intake; the blood samples were then drawn before the midweek dialysis session. The ESRD patients had lower levels of GR, GPx, and SOD activity, a lower level of FRAP, and a higher UA concentration than the control group. The FRAP value decreased with age (*ρ* = −0.32, *p* = 0.001); smokers had a significantly lower SOD activity in comparison to nonsmokers (*p* = 0.03). In the ESRD patients, FRAP and UA correlated with both albumin (*ρ* = 0.26, *p* = 0.011; *ρ* = 0.41, *p* = 0.006, respectively) and prealbumin (*ρ* = 0.34, *p* ≤ 0.001; *ρ* = 0.28, *p* = 0.006, respectively), whereas UA, GR, GPx, and SOD correlated with calcium, UA, GR, and GPx with phosphate level. Based on the findings, there are weak associations between nutritional status and selected redox parameters in hemodialyzed patients. Further studies are needed to establish if diet modifications and adequate nutritional status can positively impact the antioxidant capacity in this group of patients.

## 1. Introduction

Over 50% of patients in end-stage renal disease (ESRD) die of cardiovascular events [1]. In the general population, traditional risk factors for cardiovascular disease (CVD) include smoking, hypertension, obesity, and increased LDL or low HDL cholesterol levels [2]. Patients treated with maintenance hemodialysis frequently develop additional complications which may further exacerbate the progression of CVD. They include hypoalbuminemia, anaemia, disturbed calcium-phosphate metabolism, electrolyte imbalance, increased sympathetic nervous system activity, and metabolic acidosis [3]. Numerous studies, however, emphasize that what lies at the root of CVD pathogenesis is oxidative stress (OS). Hemodialysis patients are especially vulnerable to redox balance disruption as they are repeatedly exposed to elevated OS during regular dialysis sessions requiring the presence of a central venous catheter or an arteriovenous fistula and the use of bioincompatible dialyzers and bloodlines [4]. It has been shown that OS, by promoting renal ischemia, may decrease residual diuresis, which is important for the proper functioning of the CV system. Excessive production of reactive oxygen species (ROS), especially O^2-^, leads to nitric oxide (NO) inactivation and deficiency [5]. NO is a critical antioxidant that protects kidney function by increasing renal blood flow through blood vessel relaxation. A decrease in NO concentration is regarded as an early mechanism of atherosclerosis, as it prevents platelet aggregation and smooth muscle hypertrophy and reduces the expression of adhesion endothelial molecules [6].

Another problem frequently developed by ESRD patients which may further disturb redox balance is the malnutrition-inflammation-atherosclerosis (MIA) syndrome, which indicates the interdependence of atherosclerosis with poor nutritional status and the inflammatory component. Stenvinkel et al. [7] distinguish two types of malnutrition. The first one can be reversed with an increase in calorie and protein supply and is rarely connected with comorbidities. Hemodialysis patients, however, face many dietary restrictions, and the first type of malnutrition can easily progress into the second type characterized by inflammatory background and markedly elevated OS. Concomitant increase in resting energy expenditure, the loss of vitamins and microelements during the hemodialysis procedure, and low intake of fruits and vegetables—a prominent source of antioxidants—can thus further disturb redox homeostasis in ESRD patients [5].

Modifiable lifestyle factors (physical activity, diet, sleep duration, alcohol intake or smoking) may each influence redox homeostasis [8–13]. ROS are generated in response to environmental stressors such as cold, viral or bacterial infections, drugs, toxins, radiation, hypoxia, and hyperoxia [14]. In ESRD patients, kidney filtration is significantly impaired, which contributes to increased accumulation of uremic toxins such as indoxyl sulphate, methylguanidine, xanthine, or orotic acid and limited elimination of cytokines and waste products. Increased activity of the mitochondrial transport chain, degradation of metabolites in lysosomes, peroxisomal lipid *β*-oxidation, oxidation of ethanol in the microsomal ethanol oxidizing system, or cytochrome P-450 detoxication of xenobiotics are metabolic processes which can lead to increased production of ROS causing stress on cellular structures and inducing changes in molecular pathways [15].

ROS may induce CV pathology by damaging lipids, proteins, and DNA and forming advanced glycation end products (AGEs) which have been shown to accumulate in atherosclerotic plaques and are thought to activate monocyte infiltration through NF-*κ*B [14]. What is more, oxidized LDL can increase expression of peroxisome proliferator activated receptors (PPAR) in foam cells of atherosclerotic lesions [14].

Equally important is the fact, however, that ROS also regulate a wide range of processes in the cardiovascular system and contribute to the maintenance of cardiovascular homeostasis [16]. They can serve as signaling molecules to regulate physiological processes and act as messengers both in the extracellular environment and within cells [17–19]. At low to modest concentrations, they are considered to be essential for the regulation of cell cycle progression and proliferation, differentiation, migration, and cell apoptosis [20]. They also promote the organism's immune functions and natural defenses—leukocytes, especially neutrophils, produce oxygen radicals through NADPH oxidase in the respiratory burst to protect the host organism from pathogens [8].

ESRD patients are regularly exposed to OS during hemodialysis sessions. In the study, we investigated the relationships between redox balance in patients treated with maintenance hemodialysis and their nutritional status, inflammation, and iron and calcium-phosphate metabolism. Also, the impact of selected modifiable lifestyle factors on the redox balance was assessed.

## 2. Materials and Methods

### 2.1. Patients

The cross-sectional study included adult patients in ESRD treated with maintenance hemodialysis (3–5 h dialysis sessions three times a week). The study group was recruited at two centres: the Department of Nephrology of Jagiellonian University Medical College in Krakow, Poland and the Dialysis Therapy Centre of Clinical District Hospital No. 2 in Rzeszow, Poland in December 2016 and January 2017. The inclusion criteria were age 18 years or older, ESRD diagnosis defined according to KDIGO [21], and repeated maintenance hemodialysis treatment for a period of at least 1 month. Patients were dialyzed using an arteriovenous fistula (82%), a permanent catheter implanted in the jugular vein (15%), or an acute catheter placed in the subclavian, jugular, or femoral vein (3%). In 78% of the patients, low-flux dialyzers were used, while the remaining group underwent hemodiafiltration with high-flux dialyzers. The mean weekly hemodialysis time was 12 hours ± 30 minutes. The adequacy of the dialysis was evaluated based on clinical symptoms and the *Kt*/*V* index assessed once per month. Most patients (98.5%) had a *Kt*/*V* ≥ 1.2.

In order to assess control values of redox balance laboratory tests, a control group was recruited from workers undergoing routine periodic occupational medicine examinations whose blood samples were examined at the Diagnostic Department of the University Hospital in Krakow, Poland. These persons had GFR > 60 mL/min/1.73 m^2^, no history of renal disease, and no acute health issues, and their C-reactive protein (CRP) concentration was below 5 mg/L. They passed the routine health checkup with no new diseases diagnosed. Participation in the study was voluntary, and each patient gave his/her written consent to take part in the study. The study protocol had the consent of the Bioethics Committee of the Regional Medical Chamber in Rzeszow, Poland (approval number 70/2014/B issued on 19 September 2014).

### 2.2. Questionnaire

A self-constructed questionnaire was administered among hemodialysis patients. It was completed by the patients on their own or with the author's assistance (M.M.) whenever needed. The questionnaire gathered information on patients' physical activity (low/moderate), hours of sleep (6 hours or less/day, 7-8 h/day, 9 hours or more/day), frequency of fruit and vegetable intake (one or more portion/day, less than 1 portion/day), and smoking.

### 2.3. Laboratory Tests

In the study group, hematology and biochemistry tests including white blood cells (WBC), platelet (PLT), iron, total iron binding capacity (TIBC), total calcium, phosphate, albumin, prealbumin (PRE), and CRP were performed in the Diagnostic Department of the University Hospital in Krakow, Poland as routine monitoring of patients. Due to local differences in patient monitoring, lipid profiles (total cholesterol, LDL cholesterol, HDL cholesterol, and triglycerides) and iPTH were only available for patients treated in Rzeszow. Blood samples were collected before the midweek dialysis session.

The aliquots of serum/plasma were frozen in -80°C and used for the measurement of parameters associated with redox balance and nutritional status. In the control group, aliquots of samples drawn for the routine laboratory tests were frozen for the purpose of the present study.

The assays used to measure redox balance including glutathione reductase (GR), glutathione peroxidase (GPx), superoxide dismutase (SOD), and ferric reducing antioxidant power (FRAP) were conducted at the Department of Diagnostics of Jagiellonian University Medical College, Krakow, Poland.

GR activity was evaluated based on the reduction of oxidized glutathione (GSSG) in the presence of NADPH, oxidized into NADP^+^ [22]. GPx activity was measured based on the decrease of NADPH absorbance at 340 nm according to the Paglia and Valentine method [23].

SOD activity was assessed with the Misra and Fridovich method [24], which involves oxidation of adrenaline to adrenochrome in the basic pH environment. The measurement was conducted at a wavelength of *λ* = 480 nm. An activity unit for SOD was defined as an amount of enzyme which causes 50% inhibition of adrenaline autoxidation.

Total antioxidant capacity was measured using the FRAP method. Fe^3+^ ions in the tripyridyltriazine complex (Fe^3+^-TPTZ) were reduced by serum low-molecular antioxidants. The reaction product—Fe^2+^-TPTZ—has an intense blue color and presents maximum absorbance at wavelength *λ* = 593 nm. Antioxidant capacity was assessed through the measurement of absorbance change in the assay and the sample Fe^2+^ solution [25].

Nutritional status of the study group was assessed using body mass index (BMI), based on body mass measurements conducted after the midweek dialysis session and albumin, prealbumin, CRP/PRE, and Glasgow Prognostic Score (GPS). GPS is a useful survival predictor in advanced stages of cancer as it takes into account both inflammation and malnutrition and has been used also in ESRD. A GPS score value (0–2) was assigned based on the CRP and albumin concentrations (score = 0 if CRP ≤ 10 mg/L and albumin ≥ 35 g/L; score = 1 if CRP > 10 mg/L or albumin < 35 g/L; score = 2 if CRP > 10 mg/L and albumin < 35 g/L) [26].

### 2.4. Statistical Analysis

The number of patients (percentage of the appropriate group) was reported for categories. Contingency tables were analyzed with a chi-squared test. Mean ± standard deviation (SD) and median (lower-upper quartile) were shown for the quantitative variables with normal and nonnormal distribution, respectively. The differences between groups were assessed with the *t*-test or Mann–Whitney test, according to distribution. The Spearman rank coefficient (*ρ*) was used to study correlations as most variables were nonnormally distributed. Logistic regression was used to assess whether differences between the control and the study group were independent of sex, age, and CRP. Linear regression was used to verify whether the correlations between redox and nutritional parameters were independent of age, dialysis vintage, comorbidities, and inflammation in ESRD patients; CRP values were log transformed prior to the analysis as the variable was highly right skewed. The highly correlated variables (such as albumin and prealbumin or total and LDL cholesterol) were assessed in separate linear regression models.

Results were considered significant at *p* < 0.05. The analysis was performed with Statistica 12.0 (StatSoft, Tulsa, OK, USA) software.

## 3. Results

The study included 97 ESRD patients on maintenance hemodialysis and 42 controls (Table 1). The groups differed in age, sex, CRP, and nutritional parameters (Table 1). The ESRD patients were hemodialyzed for a period of 1.5 months to 32 years (Table 2). The lifestyle questionnaire (Table 2) showed that 17% were active smokers, and less than half declared a moderate level of physical activity. As much as 39% of the patients consumed fruits and 46% consumed vegetables less frequently than once a day. Over 63% of the patients had calcium levels below the reference range and over 80% of patients had phosphorus levels above the upper reference limit (Table 1). Total cholesterol and LDL cholesterol were above the reference range in about a quarter of the patients, and 18% had HDL cholesterol below the reference range. Fifty three percent of the patients had a TIBC below the lower reference limit (Table 1).

The studied group of ESRD patients differed significantly from the controls in all the studied redox parameters: the hemodialysis patients had lower levels of GPx, GR, and SOD activity, a lower FRAP value, and higher UA concentrations than the control group (Table 1). The differences between patients and controls in terms of GPx, GR, and FRAP values were significant after adjustment for sex and age; however, after additional adjustment for CRP, only GPx and SOD differed significantly between patients and controls (Table 3).

In ESRD patients, the FRAP values decreased with age (*ρ* = 0.32, *p* = 0.046). A longer duration of maintenance dialysis treatment was associated with higher FRAP values (*ρ* = 0.26, *p* = 0.014), GR activity (*ρ* = 0.35, *p* ≤ 0.001), and UA level (*ρ* = 0.35, *p* ≤ 0.001). No sex-related differences were observed in redox parameters in the study group. In controls, UA increased with age (*ρ* = 0.41, *p* = 0.017) and men and women differed in FRAP value (1.21 ± 0.26, 1.01 ± 0.26; *p* = 0.019) and UA level (324.8 ± 49.0, 266.8 ± 66.6; *p* = 0.013), respectively.

The studied redox parameters were intercorrelated both in the ESRD patients and in controls. In the hemodialyzed patients, there were statistically significant correlations between GR and SOD (*ρ* = −0.23, *p* = 0.02), GR and bilirubin (*ρ* = 0.39, ≤0.001), GPx and UA (*ρ* = 0.42, p ≤ 0.001), and FRAP and UA (*ρ* = 0.47, *p* ≤ 0.001). In the control group, FRAP correlated with UA (*ρ* = 0.81, *p* ≤ 0.001) and GR (*ρ* = 0.45, *p* = 0.004).

**Figure 1:**
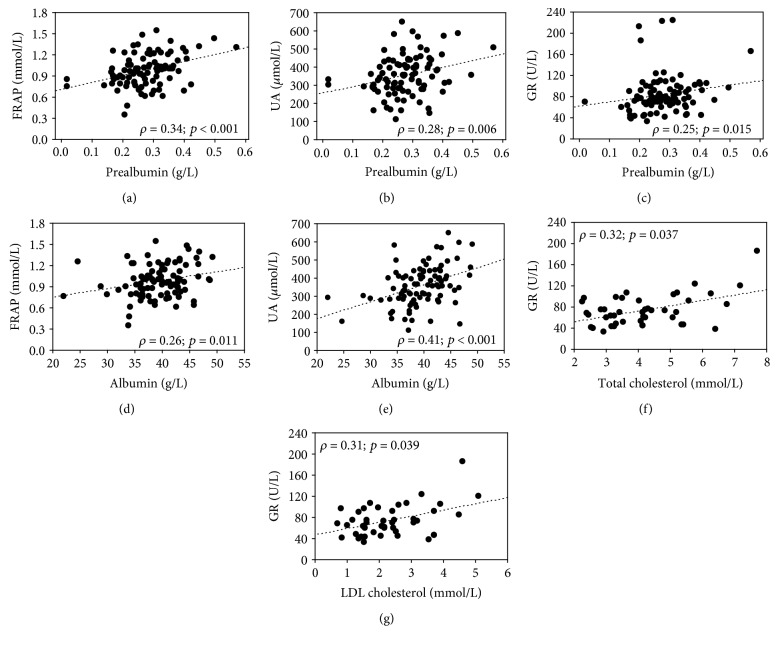
Correlations between selected redox and nutritional parameters in ESRD patients: ferric reducing antioxidant power (FRAP) and prealbumin (a); uric acid (UA) and prealbumin (b); glutathione reductase (GR) and prealbumin (c); FRAP and albumin (d); UA and albumin (e); GR and total cholesterol (f); GR and LDL cholesterol (g).

In ESRD patients, several correlations were observed between the nutritional status biomarkers and redox parameters—albumin correlated positively with FRAP and UA; similarly, prealbumin correlated with FRAP, GR, and UA. There was a positive correlation of total cholesterol and LDL cholesterol with GR (Figure 1). Of those correlations, the associations between FRAP and prealbumin, UA and albumin and prealbumin, as well as GR and prealbumin, total cholesterol, and LDL cholesterol were independent of age, dialysis therapy duration, comorbidities, and CRP of ESRD patients (Table 4). No correlations were observed between redox and nutritional parameters in the controls. Moreover, in ESRD patients, GPx inversely correlated with PLT (*ρ* = −0.41, *p* ≤ 0.001) and WBC (*ρ* = −0.27, *p* = 0.007). FRAP showed a positive correlation with Fe (*ρ* = 0.32, *p* = 0.005) and TIBC (*ρ* = 0.29, *p* = 0.012). The associations of FRAP with Fe (beta 0.29 ± 0.11, *p* = 0.008) and TIBC (beta 0.31 ± 0.11; *p* = 0.008) were both independent of age, dialysis therapy duration, comorbidities, and CRP of ESRD patients.

Significant correlations were observed between redox parameters and serum concentrations of total calcium and phosphate (Table 5). UA correlated positively with total calcium and inversely with phosphate level. SOD correlated positively with total calcium, whereas GR and GPx with both total calcium and phosphate (Table 5). No correlation was found between the redox parameters and iPTH.

The analysis of lifestyle factors in the ESRD patients showed that there were no statistically significant differences in redox parameters between patients declaring low and those declaring moderate physical activity. Patients eating at least one portion of fruit per day had a higher UA level (*p* = 0.024), similar to those eating at least one portion of vegetables per day (*p* = 0.044). No other significant differences were observed in relation to fruit and vegetable frequency intake. No differences were recorded between groups declaring different amounts of sleep daily. SOD was higher in nonsmokers than in smokers (*p* = 0.027).

## 4. Discussion

The comparison of redox parameters in the control and the study group clearly showed that hemodialyzed patients had a lower activity of endogenous enzymes and FRAP value as compared to healthy controls. The GPS, which takes into account albumin and CRP level values, showed that nearly 36% of patients had some degree of malnutrition or inflammation. Malnutrition and hypoalbuminemia reduce antioxidant defense [27], and albumin and prealbumin, commonly used nutritional markers, possess antioxidant properties and can account for significantly lower FRAP values when compared with the controls.

Moreover, FRAP increased with dialysis duration, which may imply that the increased OS to which patients are exposed during maintenance hemodialysis sessions induces a strong compensatory response of antioxidant mechanisms and reflects the organism's effort to activate an antioxidant defense. Nevertheless, the antioxidant capacity diminishes with age and redox balance appears to be more difficult to maintain.

Hemodialysis patients are subject to a number of dietary restrictions (e.g., reduction of phosphorous intake, an important component of protein foods); therefore, their overall diet frequently does not meet daily caloric and protein requirements [28]. Moreover, the risk of elevated potassium and fluid overload, which contribute to severe CV complications and increased mortality in hemodialysis patients [3], may lie behind decreased fruit and vegetable consumption in this group of patients [29]. Meanwhile, fruits and vegetables supply the organism with low-molecular exogenous antioxidants and may contribute to the overall antioxidant capacity of plasma.

An equally important element of defense against free radicals is the antioxidant system which requires specific microelements, which can be deficient in a restrictive, low-calorie diet. An inadequate intake of elements such as copper, selenium, manganese, or iodine can translate into incorrect functioning of endogenous enzymes. Extracellular GPx, for example, is a selenium-dependent enzyme, and extracellular SOD contains copper and zinc in its structure. Montazerifar et al. [30] have shown that hemodialysis patients who had higher intake of fruits and vegetables had a concomitant increase in SOD activity.

In our study, patients who consumed more fruits and vegetables had a higher UA level which could be attributed to an overall higher intake of food and high-purine products. However, several in vitro and in vivo studies have also shown that fructose, present in fruits and in moderate amounts in vegetables, increases blood UA levels [31]. Fructose per se can also induce oxidative stress as it stimulates mitochondrial metabolism leading to an increase in ROS production through an apocynin-sensitive pathway [32]. In our study, the FRAP value correlated with UA as its estimated total contribution to FRAP is 60% (protein—10%, bilirubin—5%, ascorbic acid—15%, *α*-tocopherol—5%, and other compounds—5%) [33]. UA is a powerful oxygen radical scavenger in hydrophilic environments, and a recent study on a large cohort showed that low and not high serum UA levels predicted all-cause and CV mortality [34]. Interesting is the fact that in the control group, despite lower UA, a higher FRAP value was reported. The hemodialysis patients, on the other hand, had higher UA but lower FRAP value. This would mean that in the control group, components other than UA contribute to the total antioxidant capacity of plasma, a fact which may be involved with their less restrictive diets and better nutritional status.

The analysis of correlations between the nutritional and redox parameters (Figure 1) showed several relationships, most of which have been confirmed in multiple linear regression adjusted for age, dialysis vintage, comorbidities, and CRP of ESRD patients (Table 4). Nevertheless, the correlations were weak. The most probable explanation is that many factors may influence both nutritional and redox parameters measured in serum or plasma, and we have controlled only for a part of these factors.

As for other lifestyle factors and their impact on redox parameters, patients who declared active smoking had a lower SOD activity compared to nonsmokers. The study seems to be in agreement with other studies by Naga Sirisha and Manohar [35] and Manafa et al. [36]. Smoking HD patients are more prone to oxidative tissue injury than nonsmoking subjects; therefore, smoking cessation is strongly recommended [4]. What's more, long-term cigarette smoking is also a risk factor for impaired endothelium-dependent relaxation [15].

Physical activity increases metabolic processes and free radicals are the by-product of intensified mitochondrial processes. Acute and strenuous exercise can generate an excess of free radicals [37]. However, regular moderate exercise seems to counteract oxidative stress-related detrimental changes and promote a healthy lifestyle. This idea is summarized by the concept of hormesis defined as the adaptive response seen in organisms continuously exposed to low to moderate levels of stress [38]. Under these conditions, cells develop an adaptive response, including increased expression of antioxidant genes [39]. The induction of hormesis is controlled by redox sensor pathways which, upon activation by oxidants, upregulate the antioxidant enzymatic system. For example, intense physical exercise activates the mitogen-activated protein kinase (MAPK) and the NF-*κ*B redox signaling pathways. The major targets of these pathways are antioxidant enzymes, including SOD, GPx, and GSH which contain NF-*κ*B and activator protein-1 (AP-1) binding sites in their promoters as well as responsive elements to various stimuli like proinflammatory cytokines, oxygen tension, and ROS [40]. The above study did not show any association of physical activity level with the tested parameters. The reason for that may be that ESRD patients are frequently bedridden and no strenuous exercise is advised; however, moderate activity could influence the redox homeostasis and further studies on a larger cohort are warranted.

A considerable challenge in ESRD patients is maintaining calcium-phosphate balance. Impaired elimination of phosphate by the kidneys in ESRD patients has been associated with vascular calcification and cardiovascular events as well as induction of secondary hyperparathyroidism, which is one of the causative agents of increased incidence of CV complications in the studied population [21]. Phosphate is mainly a structural component of nucleic acids, adenosine triphosphate (ATP), and the phospholipids of membranes, but it is also necessary for the phosphorylation of nicotinamide adenine dinucleotide (NAD). The reduced form (NADPH) is a unique provider of reducing equivalents to maintain or regenerate the cellular detoxifying and antioxidative defense systems; NADP^+^, on the other hand, has acquired signaling functions [40]. Recent studies have shown that elevated extracellular phosphate concentrations cause mitochondrial OS by increasing the mitochondrial membrane potential, leading to caspase activation and the subsequent induction of apoptosis [41, 42]. Alteration in the levels of extracellular inorganic phosphate (Pi) triggers signaling to regulate gene expression and cellular functions in some types of cells [43]. In extraskeletal tissues, it results in the calcification associated with the upregulation of osteoblast marker genes in vascular smooth muscle cells. An increase in extracellular Pi triggers signal transduction via the PiT1 type III sodium-phosphate cotransporter and ERK1/2 pathway [43, 44]. What is becoming increasingly apparent is that there are significant interactions between calcium and redox species, such as superoxide anion (O^2-^), hydrogen peroxide (H_2_O_2_), and hydroxyl radicals (HO^−^) and that these interactions modify a variety of proteins that participate in signaling transduction pathways and in other fundamental cellular functions that determine cell life or death [45]. Interactions can be considered bidirectional—ROS can regulate cellular calcium signaling, whereas calcium signaling is essential for ROS production [46].

The results show that there is an inverse relationship between GPx and GR and total calcium and phosphate, respectively, as well as SOD and total calcium, which shows some degree of association between endogenous enzymes and calcium-phosphate metabolism. No correlation was found between the redox parameters and iPTH. This may be due to the medication administered to patients according to KDIGO 2017 guidelines (phosphate binders, calcimimetics, and active form of vitamin D) [21] in order to limit the harmful influence of secondary hyperparathyroidism on the CV system.

### 4.1. The Limitations of the Study

Our study has several limitations. The number of subjects included may limit the ability to detect the weak associations of the systemic levels of redox parameters with the lifestyle factors. The questionnaire used to assess physical activity and fruits and vegetables included only two or three variable questions. A 24-hour dietary recall would allow for a more accurate evaluation of a patient's diet; however, our questionnaire was designed in a way that could be easily (and thus willingly) used by hemodialyzed patients. Moreover, the design of the study does not allow assessing the causality of the observed associations.

## 5. Conclusion

ESRD patients are inevitably exposed to OS during regular dialysis sessions. With longer durations of dialysis, compensatory mechanisms may be induced to maintain redox balance. Nevertheless, OS resistance diminishes with age and in consequence, redox homeostasis is significantly disturbed. Our results imply that nutritional status is associated with the redox balance, although the associations are weak and most possibly affected (or driven) by multiple factors. The lifestyle factors, such as smoking, can affect redox parameters and should be taken into account in hemodialysis patients' treatment. However, larger studies and longitudinal observation are necessary to assess whether (and to what extent) the lifestyle factors may modify the redox balance in hemodialyzed ESRD patients, and what is the causality of nutritional status-redox balance associations.

## Figures and Tables

**Table 1:** Demographic data and selected laboratory results in the ESRD patients and control group.

Laboratory parameters	ESRD patients (*N* = 97)	Control group (*N* = 42)	*p*	Reference range
Age (years)	62 ± 14	52 ± 18	≤0.001	Not applicable
Males (*n*, %)	60 (61.9)	17 (40.5)	0.023	Not applicable
Albumin (g/L)	39.4 (36.4–42.8)	44.5 (40.3–47.0)	<0.001	35–52
Prealbumin (g/L)	0.27 (0.22–0.32)	No data	—	0.20–0.40
CRP (mg/L)	5.75 (2.2–13.7)	1.45 (1.0–1.4)	≤0.001	<5
PLT (×10^3^/*μ*L)	203 ± 67.4	240 ± 66	0.005	125–340
WBC (×10^3^/*μ*L)	6.5 (5.1–7.8)	5.8 (5.3–6.8)	0.267	4–10
Total cholesterol (mmol/L)^∗^	4.2 ± 1.4	5.2 ± 0.8	<0.001	3.2–5.2
LDL cholesterol (mmol/L)^∗^	2.3 ± 1.1	2.9 ± 0.9	<0.001	0.2–3.4
HDL cholesterol (mmol/L)^∗^	1.0 ± 0.3	1.5 ± 0.4	0.022	0.9–3.0
Triglycerides (mmol/L)^∗^	1.5 (1.2–2.2)	1.9 (1.6–2.3)	0.210	0.2–2.3
Bilirubin (*μ*mol/L)	8.5 (6.8–10.3)	10.1 (8.7–17.2)	0.014	0–21
Uric acid (*μ*mol/L)	357.9 ± 107.6	287.94 ± 66.37	≤0.001	F: 143.0–340.0
				M: 202.0–416.0
Total calcium (mmol/L)	1.89 (1.18–2.27)	No data	—	2.15–2.55
Phosphate (mmol/L)	2.50 (1.65–4.50)	No data	—	0.81–1.45
iPTH (pg/mL)^∗^	174.4 (75.4–410.7)	No data	—	14.9–56.9
Iron (*μ*mol/L)	11.80 (9.12–15.00)	No data	—	5.83–34.50
TIBC (*μ*mol/L)	42.2 ± 10.3	No data	—	40.8–76.6
GPS				
0 (*n*, %)	56 (57.7)	39 (92.9)		
1 (*n*, %)	25 (25.8)	3 (7.1)	<0.001	0
2 (*n*, %)	11 (11.3)	0		
CRP/PRE	0.019 (0.008–0.0587)	No data	—	No data
Glutathione peroxidase (U/L)	75.9 ± 35.8	125.9 ± 18.8	≤0.001	No data
Glutathione reductase (U/L)	76.7 (66.7–98.3)	100.7 (91.0–111.6)	≤0.001	No data
FRAP (mmol/L)	0.88 ± 0.23	1.10 ± 0.27	0.027	No data
Superoxide dismutase (U/mL)	13.2 (9.8–17.5)	19.4 (13.6–23.9)	0.002	No data

LDL—low-density lipoprotein; HDL—high-density lipoprotein; iPTH—intact parathyroid hormone; PLT—platelet counts; WBC—white blood cells; TIBC—total iron binding capacity; GPS—Glasgow Prognostic Score; PRE—prealbumin; CRP—C-reactive protein; F—females; M—males; FRAP—ferric reducing antioxidant power. ^∗^Due to local differences in patient monitoring, lipid profiles and iPTH were only available for 44 patients treated in Rzeszow, Poland.

**Table 2:** Clinical characteristics and lifestyle factors in the ESRD patients.

Characteristic	ESRD patients (*N* = 97)
Dialysis therapy duration (months)	63 (36–144)

Smokers (*n*, %)	17 (17.5)

Comorbid conditions (*n*, %)	80 (81.6)
Diabetes mellitus (*n*, %)	25 (25.7)
Hypertension (*n*, %)	57 (58.8)
Osteoporosis (*n*, %)	22 (22.7)
Periodontal disease (*n*, %)	8 (8.2)
Autoimmunological disease (*n*, %)	4 (4.1)

Sleep	
6 hours or less/day (*n*, %)	32 (33.0)
7-8 h/day (*n*, %)	48 (49.5)
9 hours or more/day (*n*, %)	15 (15.5)

Physical activity	
Moderate (*n*, %)	44 (45.4)
Low (*n*, %)	51 (52.6)

Fruit frequency	
≥One portion/day (*n*, %)	57 (58.8)
<One portion/day (*n*, %)	36 (37.1)

Vegetable frequency	
≥One portion/day (*n*, %)	50 (51.5)
<One portion/day (*n*, %)	42 (43.3)

BMI (kg/m^2^)	
Women	24.5 ± 5.8
Men	25.7 ± 4.4

Abbreviations: BMI—body mass index; *N*—number of patients; ESRD—end-stage renal disease.

**Table 3:** Differences between ESRD patients and controls assessed by logistic regression adjusted for sex, age, and CRP. Odds ratios (OR) for being in the study group are reported with 95% confidence intervals (CI).

	Adjusted for sex and age		Adjusted for sex, age,and CRP	
	OR (95% CI)	*p*	OR (95% CI)	*p*
Glutathione peroxidase (per 1 U/L)	0.94 (0.92; 0.98)	≤0.001	0.97 (0.95; 0.98)	≤0.001
Glutathione reductase (U/L)	0.99 (0.97; 0.998)	0.021	1.00 (1.98; 1.02)	0.7
FRAP (mmol/L)	0.10 (0.01; 0.64)	0.016	0.44 (0.06; 3,14)	0.4
Uric acid (per 10 *μ*mol/L)	1.07 (1.02; 1.12)	0.011	1.01 (0.96; 1.06)	0.7
Superoxide dismutase (U/mL)	0.98 (0.95; 1.02)	0.3	0.91 (0.85; 0.97)	0.003

CRP—C-reactive protein; FRAP—ferric reducing antioxidant power.

**Table 4:** The results of multiple linear regression in ESRD patients showing the associations between the redox and nutritional parameters that were independent of covariates. Standardized beta coefficients (95% confidence intervals) and *p* values were shown for dependent variables, and the coefficients of determination (*R*^2^) were shown for the models.

Independent variable	Dependent variable					
	FRAP (mmol/L)	Uric acid (*μ*mol/L)		Glutathione reductase (U/L)		
		Model 1	Model 2	Model 1	Model 2	Model 3
Albumin (g/L)	Not included	0.45 (0.23; 0.68); *p* < 0.001	Not included	Not included	Not included	Not included
Prealbumin (g/L)	0.26 (0.04; 0.49); *p* = 0.026	Not included	0.27 (0.03; 0.51); *p* = 0.030	0.27 (0.03; 0.51); *p* = 0.032	Not included	Not included
Total cholesterol (mmol/L)	Not included	Not included	Not included	Not included	0.47 (0.17; 0.77); *p* = 0.005	Not included
LDL cholesterol (mmol/L)	Not included	Not included	Not included	Not included	Not included	0.46 (0.15; 0.76); *p* = 0.007
Age (years)	-0.24 (-0.45; -0.03); *p* = 0.028	-0.05 (-0.25; 0.16); *p* = 0.6	-0.09 (-0.31; 0.13); *p* = 0.4	0.12 (-0.11; 0.34); *p* = 0.3	0.01 (-0.29; 0.31); *p* = 0.9	0.01 (-0.29; 0.31); *p* = 0.9
Dialysis vintage (months)	0.22 (0.02; 0.42); *p* = 0.035	0.23 (0.03; 0.42); *p* = 0.026	0.23 (0.02; 0.44); *p* = 0.032	0.20 (-0.01; 0.41); *p* = 0.07	0.19 (-0.12; 0.50); *p* = 0.2	0.25 (-0.06; 0.55); *p* = 0.1
Comorbidity	0.20 (0.01; 0.40); *p* = 0.049	0.03 (-0.17; 0.23); *p* = 0.8	-0.02 (-0.22; 0.19); *p* = 0.9	-0.10 (-0.31; 0.12); *p* = 0.4	-0.13 (-0.44; 0.18); *p* = 0.4	-0.12 (-0.43; 0.19); *p* = 0.5
Log (CRP, mg/L)	0.06 (-0.15; 0.27); *p* = 0.6	0.23 (0.03; 0.44); *p* = 0.028	0.17 (-0.05; 0.39); *p* = 0.1	0.18 (-0.05; 0.51); *p* = 0.1	0.03 (-0.28; 0.33); *p* = 0.9	0.01 (-0.31; 0.32); *p* = 0.9
*R* ^2^ and *p* value for the model	0.25; *p* < 0.001	0.26; *p* < 0.001	0.16; *p* = 0.012	0.13; *p* = 0.046	0.29; *p* = 0.048	0.28; *p* = 0.064

FRAP—ferric reducing antioxidant power.

**Table 5:** Correlations between the redox parameters and calcium and phosphate concentrations in the serum of ESRD patients.

	Calcium (mmol/L)	Phosphate (mmol/L)
Glutathione peroxidase (U/L)	*ρ* = −0.29; *p* = 0.004	*ρ* = 0.25; *p* = 0.012
Glutathione reductase (U/L)	*ρ* = 0.31; *p* = 0.002	*ρ* = −0.28; *p* = 0.005
FRAP (mmol/L)	*ρ* = 0.20; *p* = 0.051	*ρ* = −0.05; *p* = 0.638
Uric acid (*μ*mol/L)	*ρ* = 0.41; *p* ≤ 0.001	*ρ* = −0.42; *p* ≤ 0.001
Superoxide dismutase (U/mL)	*ρ* = 0.24; *p* = 0.017	*ρ* < 0.01; *p* = 0.968
CRP (mg/L)	*ρ* = 0.01; *p* = 0.847	*ρ* = −0.05; *p* = 0.629

CRP—C-reactive protein; FRAP—ferric reducing antioxidant power.

## Data Availability

The questionnaire and laboratory data used to support the findings of this study are available from the corresponding author upon request.
